# Psychiatrization of Resistance: The Co-option of Consumer, Survivor, and Ex-patient Movements in the Global South

**DOI:** 10.3389/fsoc.2022.784390

**Published:** 2022-03-08

**Authors:** Jenny Logan, Justin M. Karter

**Affiliations:** ^1^Brooklyn Institute for Social Research, Brooklyn, NY, United States; ^2^Counseling Services, Boston College, Boston, MA, United States

**Keywords:** psychiatrization, sexual violence, consumer/survivor/ex-patient, discipline, MGMH

## Abstract

This article examines contemporary examples of psychiatrization as a tool of disciplinary control and repression, focusing on new research on the co-option of consumer/survivor/ex-patient movements within the Global South. Here, we understand psychiatrization as (1) the process of imposing certain interpretive limits on states of difference and distress and (2) the conceptualization of treatment and recovery through the teleological notion of normalcy. By interpreting difference solely in psychiatric terms, psychiatrization functions as a tool of disciplinary control in both domestic and international contexts by reterritorializing efforts to resist hegemonic norms and political institutions of gendered and racialized oppression, colonialism, and imperialism. After setting out our understanding of psychiatrization as a political process in the sense that it enacts a particular “ontological politics”, one that foregrounds psychiatric interpretations of difference and dissent to the exclusion of other possible meanings, we examine the reach and complexity of psychiatrization in the suppression of political and social movements that attempt to resist oppressive norms and institutions. We then present new research within the consumer/survivor/ex-patient and psychosocial disability movements in the Global South to show how psychiatrization can thwart activist's aims of transforming how we view both the end goals of mental health treatment and the political valence of mental distress.

## Introduction

In the introduction to *Red, White*, and *Black*, Wilderson (2010) describes an Indigenous man who sits on Telegraph Avenue in Berkeley with a sign requesting payment for stolen land, and a Black woman who yells at students passing by her Harlem doorstep for stealing her couch and for selling her into slavery (p. 1). As Wilderson remarks, the Black woman and the Indigenous man must be constructed as “crazy” to support our continued avoidance of difficult questions about reparations, land, and justice. We argue that the psychiatrization of attempts to resist oppression and seek justice is a significant way in which “mental illness” is deployed to manage and control marginalized populations, including and especially people of color and people living in poverty. Here, we understand psychiatrization as (1) the process of imposing certain interpretive limits on states of difference and distress and (2) the conceptualization of treatment and recovery through the teleological notion of normalcy. By interpreting difference solely in psychiatric terms, psychiatrization can function as a tool of disciplinary power in both domestic and international contexts by reterritorializing efforts to resist hegemonic norms and political institutions of racial capitalism, colonialism, and imperialism.

Drawing upon Mol (1999)'s conception of ontological politics—which refers to practices of framing problems and producing bodies within the construction of a social problem—we understand the psychiatrization of difference from and resistance to hegemonic norms as a political-ontological process. This paper proceeds in three parts: first, we argue that psychiatrization can function as a process of disciplinary control by enacting an ontological politics that foregrounds psychiatric interpretations of difference and dissent to the exclusion of other possible meanings. Here, we use “disciplinary” in a Foucauldian sense to refer to the production and reproduction of subjects through tactics of control that serve the ends of power (Foucault, 2003). We are specifically concerned with psychiatrization as a disciplinary tactic insofar as it shores up the racialized, gendered, and ableist hierarchies intrinsic to global capitalism (Ben-Moshe, 2020).

Second, we examine several tension points between psychiatrization and political and social movements that attempt to express resistance to hegemonic norms and exploitative institutions, to give a sense of the complexity and global reach of processes of psychiatrization that function as a form of disciplinary control over participants in these movements. We then present new research on the institutional co-option of the consumer, survivor, and ex-patient movements within the Movement for Global Mental Health (MGMH), to show how processes of psychiatrization thwart activist's aims of transforming how we view both the end goals of mental health treatment and the political valence of mental distress caused by social and political conditions. We conclude that psychiatrization's disciplinary function constitutes an impediment to psychiatry's larger goal of alleviating suffering caused by mental difference and distress, insofar as psychiatrization neutralizes political resistance to the very institutions and norms that cause distress.

## Psychiatrization as Ontological Politics

As Laclau (1981/2021) observed, politics is “the construction of the unthinkable;” it can also, as Foucault noted, constitute a continuation of war with “other means” (Foucault, 2003). The science of psychiatry attempts to diagnose, prevent, and treat mental disorders. Modern psychiatric classification systems such as the Diagnostic and Statistical Manual of Mental Disorders (DSM) were born of pragmatic needs: to meet the demands of the growing field of medical statistics in the 20th century, clinicians needed descriptive and operationalizable criteria for sorting patients (Aftab and Ryznar, 2021). The Movement for Global Mental Health, formed in 2007, represents a growing attempt to measure, prevent, and treat mental disorders worldwide, largely by importing the techniques and classificatory systems of Western psychiatry (Mills, 2014). While the MGMH contains a diversity of viewpoints on culture and mental health, with some acknowledgment of the need to adapt methods and interventions to specific contexts, the thrust of this movement assumes that the current conceptual, diagnostic, and treatment approaches of psychiatry can be applied without engaging ethnographic and anthropological work and without critical reflexive analysis of the evidence-base of the Western mental health field (Kirmayer and Pedersen, 2014; Beresford, 2018). Across the reports and publications produced by different players in the MGMH, including academic groups and large international development organizations, the framing of mental distress ranges from categories such as “mental health problems,” “mental illness,” and “mental disorders” (De Silva and Roland, 2014), to “behavioral, developmental, and neurological disorders” (United Nations General Assembly, 2015).

Like the processes of framing mental distress utilized within the MGMH, the psychiatrization of mental distress can be a political process in at least two senses: first, it constrains the ways in which we conceive of mental differences—it constructs what is “thinkable” with respect to symptoms of mental distress by limiting our interpretive apparatus to dysfunction and pathology. Acts of resistance, when conceived as pathology, force people with legitimate, counterhegemonic political and social claims—especially Black, Brown, and Indigenous peoples—into the Prison Industrial Complex and other carceral institutions (Ware et al., 2014, p. 166; Ben-Moshe, 2020). In Canada, for example, resistance to attempted assimilation and colonization forced many Indigenous people into psychiatric treatment facilities and prisons (Ware et al., 2014). Incarceration and its logic also extend beyond carceral institutions and into communities through, for example, chemical incarceration by forced medication and surveillance in state-mandated outpatient treatment (Ben-Moshe, 2020)^1^.

Second, psychiatrization can be a political process insofar as it is influenced by both private and public actors who assert political power to define notions of normalcy and public order that are consistent with or promote their own interests and values. As Mills (2015) has pointed out, for example, the use of psychiatric diagnostic categories to classify the manifestation of “symptoms” of poverty and oppression can constrain our understandings of structural factors that contribute to inequality in the first place. The use of psychiatric definitions to label complex socioeconomic phenomena, in turn, constrains our notions of pathology and recovery in the mental health context. By defining abnormality and pathology in terms of economic burdens under a capitalist theory of human value, for example, political actors who shape mental health discourse uphold a particular political and economic ideology. Reports and publications produced by different players in the MGMH, including academic groups and large international development organizations, have framed mental disorders as “highly prevalent, accounting for a large burden of disease” (Mills, 2018, p. 849).

Claims that mental disorders account for “a large burden of disease” such as those made by the MGMH have their roots in attempts to utilize the measures and metrics of physical illnesses for the calculation of statistics related to mental disorders through epidemiological data (Bemme and D'souza, 2014). Starting in 1991, the World Bank and the World Health Organization (WHO) initiated the Global Burden of Disease studies (GBDs) in an attempt to quantify the role of medical interventions in economic development and to assess progress toward them (Murray and Lopez, 1996). One of the indicators utilized by the GBDs to compare different disease categories is the Disability-adjusted Life Year (DALY) metric, which calculates how many years of life are lost to a disease category due to early death or loss of functional abilities from disability. The 2010 GBD study included “mental, neurological, and substance use disorders,” and a key finding was the rapid increase in non-communicable diseases in low- and middle-income countries (LMICs), with the proportion of the burden attributable to these diseases rising from 36% in 1990 to 49% in 2010 (Murray et al., 2012; Charlson et al., 2015). As alluded to earlier, these statistics assume (and reproduce the assumption) that categories of mental distress, such as depression, anxiety, schizophrenia, etc., apply universally across different cultures and locales.

Similarly, in a study for the World Economic Forum, Bloom et al. (2011) attempted to calculate the economic cost of mental disorders, finding that the global cost of these disorders would reach US$6 trillion by the year 2030, constituting a large percentage of all lost output and productivity worldwide. A 2016 study estimated that without the implementation of psychiatric treatments worldwide, depression, and anxiety disorders would cost the 36 largest companies in the world US$925 billion every year (Chisholm et al., 2016, p. 419). These framings of the problem of mental suffering in terms of monetary cost suggests that we conceptualize recovery only in terms of a state of affairs that restores the subject to her estimated potential for economic productivity. At the same time, institutions and disciplines devoted to treating mental health emerge as revenue-generating industries, which Ben-Moshe (2020) and others have described as the “carceral industrial complex.”

Even when assuming the cross-cultural appropriateness of using Western diagnostic constructs in non-Western settings, epidemiological analyses have criticized the GBD studies for the value judgments inherent in DALY metrics, the low quality of data in LMICs without robust health surveillance systems, and the uncritical use of the GBD estimates in academic studies and policies (Brhlikova et al., 2011). In the case of depression estimates, the GBD data were generated using a wide range of different measures and scales, which often did not allow for the use of clinical judgement in screening or diagnosis. The most common depression measures used in the GBD study, the Diagnostic Interview Schedule (DIS) and the Composite Diagnostic Interview Schedule (CIDI), are highly standardized and structured interviews, often conducted by laypeople (Brhlikova et al., 2011). Almost the entirety of validation studies for the CIDI were completed in Western samples, and its cross-cultural reliability and validity have been challenged (Ferrari et al., 2013). Moreover, GBD data from LMICs in South-East Asia and Africa was often not based on nationally representative samples and was extrapolated from studies from a small area, or even a single village (Brhlikova et al., 2011).

Further, in a much more straightforward way, members of the psychiatric profession contribute to disciplinary efforts by participating in policing. Policing refers to activities performed by “an institution that is empowered by the state to inflict social control and reinforce oppressive social and economic relationships” (Klukoff et al., 2021, p. 460). Psychiatrists participate in policing in a number of ways: conducting evaluations in civil commitment hearings that constitute evidence needed by state prosecutors to meet the “clear and convincing” standard for conservatorships, involuntary hospitalizations, and forced medication treatment [see, e.g., Addington v. Texas, 441 U.S. 418 (1979)]; making findings regarding a patient's medication compliance that can justify rejection of an applicant's petition for social security or disability benefits (see Social Security Regulation 18-3p, 2018); and performing risk assessments that are used to justify both immigration detention and incarceration for individuals awaiting trial, often using metrics that are racially biased or, at best, lacking in validity with non-white populations (see Murray and Lopez, 1996, pp. 261–62).

Both government policy and industry funding shape the discourse and practice of diagnostic psychiatry domestically and in rising initiatives in the MGMH. This private-public partnership between industry and state power, as Obert (2018) observes, continues to shape both organized violence and criminal justice in the U.S. (p. 5). Such partnerships “characterize contemporary forms of governance following the neoliberal turn” (Mulla, 2014, p. 225, citing Bumiller, 2008) and shape the delivery of psychiatric services in private hospitals, which become designated agents of the state. In this paper we show how politically and institutionally led GMH interventions such as widespread and mandated depression screening frame mental suffering as an economic burden while downplaying “concerns about neocolonialism and the ethnocentric quality of the instruments” used to measure mental health (Cosgrove and Karter, 2018, p. 674).

In the U.S., we argue, neoliberal capitalist politics inform and shape public health discourse and practice that frequently result in pro-industry policies with only passing regard to public health effects. As Jill Fisher observes, the logic of neoliberalism dictates that “What's good for the industry is good for America” (Fisher, 2007, p. 65). Here, businesses are political actors, and concentrations of market power essentially privatize political power to serve the interests of increasing market share for a few global corporations. Corporations are also political in the sense that they are products of state actions whereby the state grants certain privileges, which the Supreme Court recognized as early as 1837 in the Charles River Bridge v. Warren Bridge decision. Markets and market actors are therefore not “natural” but constructed by law, and the construction of markets by law is always political. The current economic and political order, which privileges economic “efficiency,” is also not neutral: it enacts a principle of accession that increasingly concentrates economic power through, for example, the operation of credit markets, legal rules regarding inheritance, and tax policies that favor the wealthy. Economic power is, in turn, inextricably connected to systems of unequal power that produce racialized, gendered, and ableist hierarchies (Ben-Moshe, 2020).

Whether funded by government, non-profit foundations, or pharmaceutical companies, psychiatric research is inflected with financial bias, and financial relationships with industry create “pro-industry... habits of thought” (Lexchin and O'Donovan, 2010, p. 643). Indeed, in addition to lobbying and providing direct “user fee” payments to the Food and Drug Administration in the US, pharmaceutical companies contribute funding to individual psychiatrists, medical schools, research institutions, patient advocacy groups, and politicians (see also Rose et al., 2017; Butler and Fugh-Berman, 2020). The legal and market structures that sustain this flow of capital and influence are distinctly political; as Tarek Younis succinctly puts it, “[p]olitics cannot be disassociated from public health” (Younis, 2021a,b, p. 2).

As Beeker et al. (2021) note, psychiatrization can be “top-down” in the sense that industry, governments, and other “experts” can initiate and help to normalize processes of psychiatric classification and diagnosis. While the political nature of top-down processes of psychiatrization are the most visible, bottom-up psychiatrization—psychiatrization led by individuals and group struggles for recognition of their subjective experiences of suffering in terms of psychiatric classification—is also political in the sense that it constrains how forms of mental suffering is “thinkable,” and in the sense that it is influenced by political actors both public and private. For example as Davis (2021) explains in his recent work, *Chemically Imbalanced*, private actors experiencing mental challenges often adopt medicalized explanations of their own suffering in order to avert both real and perceived allocations of blame for non-normative or “excessive” emotional responses to life events. The impulse to categorize suffering in terms of what Davis calls the “neurobiological imaginary” of modern psychiatric discourse in order to achieve a hegemonic notion of viable selfhood works to naturalize the social norms of racial capitalism. As Davis writes, these norms “are built directly into the medicalized language, [and therefore] any recourse to that language [of psychiatry] cannot but reify the social norms as the natural and inevitable yardsticks of health” (2020, p. 181).

As Fisher (2008) and Spade (2020) have argued, when forms of mental suffering have political and economic causes, collective movements to understanding those causes can lead to revolutionary action. In other words, some forms of mental suffering can engender dissent; yet both top-down and bottom-up psychiatrization processes drain suffering of its political and social contents by converting it into an intra-individual problem. The understandable impulse to eradicate feelings of sadness, loneliness, or discontent through psychiatric interventions simultaneously legitimizes valuations of mental differences in terms of economic costs and costs to productivity. As Sara Ahmed (2007/2008) has argued, “it is the very assumption that good feelings are open and bad feelings are closed that allows historical forms of injustice to disappear” (p. 135). In “treating” the suffering patient, psychiatry converts “bad” feelings into “good” ones, and these “conversions function as displacements of injury from public view” (id., p. 134). Further, the neoliberal focus on individualization effectively de-genders and de-racializes social problems (see Barad, 2007). This is not to suggest that people experiencing mental distress are not in need of care—rather, it means that viewing mental distress solely through the lens of psychiatry functions to first silo and then resignify symptoms of distress, effectively neutralizing resistance and maintaining legitimacy of the current political-economic order. To highlight the complexity and global reach of processes of psychiatrization, we provide three examples in which processes of psychiatrization territorialize distress as apolitical in the US, UK, and international contexts, before turning to new research on consumer, survivor, and ex-patient movements in the Global South and their attempts to resist processes of psychiatrization within the MGMH.

## Example 1: Protest, Rape, and Sexual Violence in the US

The construction of Black men as psychotic for speaking out against racial injustice during Civil Rights movement, and the subsequent overdiagnosis of Black men with schizophrenia, is a clear example of the repressive and disciplinary potential of psychiatrization (Metzl, 2009). As Metzl writes, “diagnostic terminology [for mental illness] is inherently politicized,” incorporating racially and politically inflected terminology (Metzl, 2009, p. 197). He continues:

“Race impacts medical communication because racial tensions are structured into clinical interactions long before doctors or patients enter examination rooms. To a remarkable extent, anxieties about racial difference shape diagnostic criteria, health-care policies, medical, and popular attitudes about mentally ill persons, the structures of treatment facilities, and, ultimately, the conversations that take place there within” (p. xii).

Top-down and bottom-up processes of psychiatrization of survivors of rape and sexual violence provides another pertinent example of the potential depoliticizing and disciplinary function of psychiatrization in the US. Anti-violence movements spearheaded by feminists of color in the 1960's and ‘70s linked gender-based violence to state violence and harms caused by public policy, structural inequality, institutionalized racism, and patriarchal power (Richie, 2012; Taylor, 2017). Movements like the Combahee River Collective in Boston, for example, recognized sexual violence and patriarchal domination within the Black community as stemming from white imperialist culture (Bryan et al., 2018). Similarly, Davis (1983) linked practices of slavery and the abuse of Black women and girls with the rape of white women:

“Once white men were persuaded that they could commit sexual assaults against Black women with impunity, their conduct toward women of their own race could not have remained unmarred. Racism has always served as a provocation to rape, and white women in the United States have necessarily suffered the ricochet fire of these attacks” (p. 177).

Understanding our shared histories of racialized violence, and our respective roles within it, is thus a crucial step in the task of interrogating the cyclical reproduction of rape and sexual abuse in America.

Yet as Bumiller (2008) has carefully documented, the feminist movement against sexual violence was gradually co-opted by the neoliberal state and used to legitimize and expand state surveillance and mass incarceration. Processes of psychiatrization have been central to this process, both in processes of converting perpetrators into pathological subjects in need of reform and deserving of criminal punishment—the “homosexual,” for example, and later the “pedophile” (see Lancaster, 2011; Harkins, 2020)—and by converting the suffering caused by sexual violence into intra-individual pathology or dysfunction. These processes have served to further legitimize both the carceral state—which disproportionately harms people of color and non-gender-conforming peoples—and the pharmaceutical industry. Psychiatrization of both victims and perpetrators of sexual violence also leaves socially marginalized women more vulnerable to violence, because women of color tend to be further harmed by psychiatric institutions^2^ (Bumiller, 2008; Metzl, 2009). Further, an essential part of the depoliticization of sexual violence was psychiatry's conceptualization of the “sex offender” as a pathological individual divorced from social and political logics. This trope has been used to rationalize expansion of state systems of surveillance and punishment, which disproportionately affect people of color.

As Harkins (2009) and Serisier (2018) have observed, individual experiences of sexual abuse have been commodified within an industry of survivor narratives that interprets these experiences as apolitical personal stories. Psychiatrically informed discourses of wellness, mental health, and self-help have played an important role in restricting the scope of meaning of sexual violence narratives to the realm of the personal, rather than the political (Serisier, 2018). Processes of psychiatrization have thus come to constitute “boundary-drawing practices” that refigure sexual violence as an apolitical phenomenon (Beres et al., 2009, p. 206), function as a disciplinary tactic by legitimizing state violence in the form of heightened surveillance and expansion the carceral state, and providing an industry solution to suffering caused by what for many activists and feminist of color can be read as a social and political problem (see Serisier, 2018).

## Example 2: Refugees, Soldiers, and the International “War on Terror”

As Howell (2011) argues, psychiatrization can have distinctly political functions in the global context as well, informing everything from the treatment of refugees and military troops to the discipline of “anti-terrorism.” Efforts of the World Health Organization and the United Nations to marshal the mental health of refugees, for example, are “aimed not only at alleviating trauma, but also at restoring order” (Howell, 2011, p. 3). The design and implementation of programs specifically for “refugees” also relies on and “reproduce[s] the notion of a system of discrete sovereign states—a system that produces statelessness and the category of refugees in the first place” (id.). Psy disciplines are increasingly implicated in the functioning and maintenance of Western militaries for the management of traumatized soldiers (id., 4). This is evidenced by steep increases in psychiatric prescription practices for soldiers and military veterans after 2001, despite simultaneous denials by the US military to confirm diagnoses and provide care to soldiers and veterans. Finally, the psy disciplines are marshaled in rendering intelligible the West's “enemies” in the war on terror, to characterize suicide bombers in terms of psychological states as opposed to political demands and motivations (id., p. 7). Kolb (2020) has traced the characterization of terror and terrorism in apolitical language—as an epidemic, for example—to the Indian Mutiny of 1857. Contemporary constructions of “terrorism” after 9/11, as Stampnitzky (2013) argues, enact a “politics of anti-knowledge” constituted by concerted efforts to deny any political understanding or rationale could be ascribed to terrorism.

## Example 3: “Radicalization” in the UK

Further, as Younis (2021a) has argued, psychologization functions as a foil that allows nation-states to evade charges of institutional racism in their management and policing of Muslims. In the UK, the human rights organization Medact.org (2021) has shown that mental health professionals collude with counterterrorism police officers to create “Vulnerability Support Hubs” to evaluate individuals suspected of “extremism.” As Medact reports, these “Hubs” “use sub-diagnostic thresholds and risk pathologizing people based on political expression or socioeconomic vulnerability” (id.). Here, psychologization's purported colorblindness effectively disguises the nation-state's racialization of Muslim populations. Further, it diverts attention from what is essentially an effort to manage of anti-hegemonic political expression by constructing racialized Muslim individuals as “at risk” or vulnerable to “radicalization.” An additional example is PREVENT, a national policy directed against radicalization in the UK, which mandates that certain public bodies evaluate individual risk factors in the “war on terror.” Like the psychiatrization of suicide bombers, the use of psychiatric discourses to justify racist policies also rules out larger political questions connected to resistance, dissent, and discontent. Indeed, as Kundnani has argued, the rise of discriminatory and racialized “risk management” practices targeting asylum seekers, “radicals,” and “Islamic terrorists” are a function of the fact that “the great well of human despair, rooted in poverty and powerlessness, can no longer be contained within national boundaries” (Kundnani, 2007, p. 1). Yet these expressions of discontent are refigured as apolitical within psychiatric discourse.

Just as top-down psychiatrization policies like PREVENT can preserve the legitimacy of a political and economic order while deploying racialized tropes and surveillance tactics against minorities, bottom-up processes of psychiatrization, as in the treatment of rape survivors described above, can also function as repressive, disciplinary tactics insofar as the language of psychiatry constrains our interpretations of violence and suffering. Indeed, as the following example from research within the consumer, survivor, and ex-patient and psychosocial disability movements show, C/S/X activists in the Global South have attempted to push back against psychiatrization but are actively discouraged and coopted by powerful actors in the MGMH.

## Co-Option of Consumer, Survivor, Ex-Patient, and Psychosocial Disability Movements

Scholars and activists with lived experience of mental distress, broadly organized under the consumer, survivor, and ex-patient (C/S/X) movement, have acted as a force against psychiatrization by opposing the medicalization of their experiences (Jones and Brown, 2012). Following the adoption of the Convention on the Rights of Persons with Disabilities (CRPD), regional groups of people with lived experience have begun to organize politically to contest psychiatrization and its effects on persons with psychosocial disability (Davar, 2008). As a recent study of the experiences of psychosocial disability advocates in the Global South (Karter, 2021) demonstrates, however, these efforts are often co-opted by powerful actors in the movement for global mental health (MGMH).

As Mills (2014) and others (see, e.g., Bhatia and Priya, 2021) have shown, the MGMH as facilitated through the World Health Organization and an assortment of NGOs has functioned as a pathway to psychiatrization of more and more populations throughout the Global South, imposing concepts of Western psychiatry upon diverse groups of people including refugees and victims of religious persecution, without regard for the nuanced regional and ethnic contexts and histories that contribute to war, violence, and mental distress. Following the ratification of the CRPD and the development of regional groups organizing under the psychosocial disability framework, psychosocial disability organizations began to be invited to participate in global mental health projects throughout the Global South. People with lived experience have often found, however, that this participation or representation was not the same as the “meaningful and authentic engagement” they were seeking (see, e.g., Russo and Wooley, 2020).

A recent qualitative study of interviews with psychosocial disability advocates in the Global South (Karter, 2021) shows how discourses of psychiatrization can operate through structural and interpersonal power dynamics to stifle resistance. Participants that were interviewed described a number of practices by MGMH groups that created barriers to the full inclusion of people with psychosocial disabilities in decision-making processes. These ranged from subtle put-downs to what appeared to be deliberate attempts to “tokenize” and “co-opt” their contributions (p. 91–96). Several activist participants described having the experience of feeling stuck when deciding between whether to engage in certain projects or to remove themselves and their organizations entirely. Participants feared that participating risked lending a sort of legitimacy to a project they did not agree with, by giving the appearance that it included lived-experience perspectives, but it could also allow them to have some influence on removing the parts they found most dangerous. On the other hand, if they refused to participate, it could send a message that these projects need to be more inclusive from the start, but it risked allowing a project to move forward that would perpetuate psychiatrization without regard to psychosocial disability advocates' political concerns (p. 93–94). One participant is quoted, describing this experience as “tokenism,” saying that “in these institutionalized spaces people with psychosocial disabilities are seen only as an endorsement. They care about our testimony, not our participation in any active way that could lead to transformation.” He added, however, that “the lack of alternatives forces us to take advantage of any space that is open to make change, to transform” (Karter, 2021, pp. 94).

While the opportunity to collaborate on new projects within the MGMH risked tokenization, C/S/X participants who had developed alternative interventions to psychiatric and psychiatrizing systems found that allowing professional psychiatrists to collaborate on these interventions risked “co-option” (Russo and Wooley, 2020). In response to these attempts to maintain the original psychiatrization approach of the MGMH, organizations of people with psychosocial disabilities and C/S/X scholar-activists have explicitly addressed the risks inherent in collaboration unless the power of the psychiatric narrative is upended. In an open letter, several such advocacy organizations—European Network of (Ex-)Users and Survivors of Psychiatry (ENUSP), Absolute Prohibition Campaign, Center for the Human Rights of Users and Survivors of Psychiatry (CHRUSP), Red Esfera Latinoamericana de la Diversidad Psicosocial, TCI Asia Pacific, and World Network of Users and Survivors of Psychiatry (WNUSP)—declared that the paradigm shift necessitated by the CRPD meant diminishing the social power of psychiatry.

Based on a social model of disability, the UN CRPD and the CRPD Committee's guidance offer us an important prospect to shift away from the biomedical paradigm when approaching madness and distress and explore not only dignified but also socially responsible and good-quality responses to human crises. This requires the relinquishment of power by the psychiatric profession and a re-definition of psychiatry's role in society. At times of such a significant historical turn, rather than admit its many failures and join efforts to collaboratively develop different and better responses, the [World Psychiatric Association] has chosen to expand its “expertise” into the field of lawmaking in order to “save the CRPD from itself” [European Network of (Ex-) Users Survivors of Psychiatry (ENUSP), 2019, p. 5].

The World Psychiatric Association (WPA) is psychiatry's global association and has taken an oppositional stance to rights-based approaches to psychiatric treatment. In the context of the MGMH debates, they have issued public statements challenging the call for rights-based approaches. The tension between proponents of the CRPD and rights-based approaches on one side and entrenched psychiatric and pharmaceutical interests on the other, has also played out through public statements and in medical journals. For example, the Australian and New Zealand Journal of Psychiatry, featured a point/counterpoint between researchers who supported the rights-based focus in mental health policy and practice, including Gill (2018) and Cosgrove and Jureidini (2019), and those who dismissed the approach as “anti-psychiatry” (see e.g., Dharmawardene and Menkes, 2018). In addition, when a special issue of the journal World Psychiatry (the official journal of the WPA) featured several articles calling for the CRPD to be amended, particularly to preserve forced treatment (see e.g., Appelbaum, 2019), six organizations of people with psychosocial disabilities issued the open letter quoted above [European Network of (Ex-) Users Survivors of Psychiatry (ENUSP), 2019].

Due to the inherent power imbalances at play in the MGMH, Russo and Wooley argue that survivor-advocates cannot join alliances or work toward change with psychiatrists: “In our view, the CRPD came about not as a demand to change psychiatry but rather as a clear call to change policies, practices, and mindsets that create psychiatry” (Russo and Wooley, 2020, p. 155). To their point, the framing of “experts and patients” inherent in psychiatric discourse can serve to undermine the rights of service-users. An analysis of the 2007 Mental Health Act in the UK, for instance, found that experts and doctors were seen as trustworthy while patients were seen as dangerous and non-compliant, severely limiting their ability to have their testimony heard and believed (Kent et al., 2020).

To move away from psychiatry's historical connection to maintaining social control in the interest of colonial powers (Hickling, 2020) that preserves and expands the global system of racialized capitalism, scholars have argued that the MGMH should adopt “a ‘pluralistic view of knowledge' that recognizes multiple voices and sources of knowledge and avoids the ‘epistemic injustice' that occurs when the knowledge of one group is validated while others are denied legitimacy” (Bemme and Kirmayer, 2020, p. 8). Given the principles of full and effective inclusion supported by the CRPD, psychosocial disability advocates may be well-positioned to contribute to this pluralistic view of knowledge, drawing upon their lived experience to bring attention to the nuances of cultural experience and contextual factors. Psychiatrization, as we have explored here, can be antagonistic to such a pluralist view of knowledge because it imposes limits on the ways in which consumers, survivors, and ex-patients are permitted to interpret their own lived experiences.

## Conclusion: Decoding Psychiatric “Illness”

*We must convert widespread mental health problems from medicalized conditions into effective antagonisms. Affective disorders are forms of captured discontent; this disaffection can and must be channeled outwards, directed toward its real cause, Capital*.—Fisher (2008, p. 80).

Psychiatrization, as we have argued, can function as an apparatus of disciplinary control that produces resistant subjects as aberrant and in need of psychiatric treatment. While some mental differences have the potential to rupture and dislocate political ideologies of gendered, ableist, and racialized oppression, exposing their contradictions and cruel logics of exploitation, psychiatrization functions to neutralize this potential. As we have argued, processes of psychiatrization can and do thwart revolutionary possibilities through the exercise of disciplinary power: through categorization, institutionalization, and chemical incarceration, and by constructing mental suffering as thinkable only in the limited ontology of the neurobiological imaginary.

As Gherovici (2003) argues in her work on the so-called “Puerto Rican syndrome,” a culture-bound diagnosis that affected working-class Puerto Rican soldiers conscripted into the U.S. military, symptom profiles that come to be understood as psychiatric illness may in fact be complex, somaticized forms of communication (see also Leader, 2011). Remarking on the often dramatic and shocking symptom profile of Puerto Rican syndrome, she writes:

“It is as if those extravagant manifestations that entered medical records were in fact messages, at times opaque, neither comprehended nor controlled by the subject” (Gherovici, 2003).

Psychiatrization limits how we read these messages; it provides a decoding key of sorts that privileges a particular kind of interpretation, one that is always already political in that it is informed by economic and ideological forces beyond the control of the well-intentioned clinician.

An ontological politics that instead affords a reading of at least some mental suffering as “forms of captured discontent” (Fisher, 2008) in addition to apolitical or irreducible distress allows us to imagine more than one modality of explanation, treatment, and recovery. This affordance can refocus our attention onto the sociopolitical conditions elided and excluded by psychiatric discourse; restore the political valence of mental difference as a challenge to the legitimacy of dominant economic, political, and social orderings; and turn our attention to the facets of political and social life that require collective action and transformation. As such, mental suffering read as manifestations of dissent might “hint at and embody aspirations that are wildly utopian, derelict to capitalism, and antithetical to its attendant discourse of Man” (Hartman, 2008, p. 12). A properly ethical interpretation of these manifestations sees “both a reconstruction and a manifestation … staged as a provocation, a call for attention, still awaiting the right decoding: … ‘Here I am, without my understanding of what it means” (Gherovici, 2003).

## Data Availability Statement

The original contributions presented in the study are included in the article/supplementary material, further inquiries can be directed to the corresponding author/s.

## Author Contributions

JL conceived the idea for the paper and wrote the first draft. JK added the section on consumer/survivor/ex-patients and contributed to both writing and editing of the finished version. All authors contributed to the article and approved the submitted version.

## Conflict of Interest

The authors declare that the research was conducted in the absence of any commercial or financial relationships that could be construed as a potential conflict of interest.

## Publisher's Note

All claims expressed in this article are solely those of the authors and do not necessarily represent those of their affiliated organizations, or those of the publisher, the editors and the reviewers. Any product that may be evaluated in this article, or claim that may be made by its manufacturer, is not guaranteed or endorsed by the publisher.

## Footnotes

^1^The development of psychiatry as a medical science is strongly linked to capitalism, capitalist expansion, and colonialism (see Mills, 2014; Ben-Moshe, 2020). Because relations of capitalist accumulation and the exclusions necessitated by imperialist expansion also produce exploited, disabled, distressed, and disenfranchised peoples, capitalist states must find ways to manage these populations. Incarceration and institutionalization are two means of “population control” that are also generative of capital through, for example, private prisons, hospitals, and security contracts (Spade, 2013, p. 1031; Ben-Moshe, 2020). Pathologizing symptoms of mental distress also has a long history in the management of capitalism's crises, as it both produces new means of capitalist accumulation—through profitable institutions like psychiatry, mental hospitals, and prescription drug manufacturing—and reduces the chances of organized political and social responses to the mental suffering caused by capitalist exploitation and economic instability (Monbiot, 2018). In British East Africa, for example, “medicalized explanations for dissent” were “far preferable [for colonizers] to economic and political analyses that might find colonial practices to be culpable in African unrest” in the early 20th century (Mahone, 2006, p. 250). The DSM has even linked unsatisfactory job performance with mental dysfunction, and in the mid-1980s, government employee assistance programs expanded to cover mental distress as a means to increase worker productivity (Davies, 2017). Incarceration, institutionalization, and psychiatrization of mental distress can all be viewed as tactics of both public and private actors to manage capitalist exploitation and its discontents while also generating profits (Gilmore, 2007). It is worth noting that these tactics engender both top-down and bottom-up processes of psychiatrization, because populations are encouraged and even required to translate their symptoms of distress into psychiatric language and new forms of psychiatric knowledge (see Lancaster, 2011).

^2^As Dean Spade argues, well-intentioned legal reform movements often leave behind the most vulnerable, centering a universal (white, female) subject of rights at the expense of others (Spade, 2013). These movements “tend[] to provide just enough transformation to stabilize and preserve status quo conditions” of racial neoliberalism (2013, p. 5).
